# Biomechanical simulation of thorax deformation using finite element approach

**DOI:** 10.1186/s12938-016-0132-y

**Published:** 2016-02-06

**Authors:** Guangzhi Zhang, Xian Chen, Junji Ohgi, Toshiro Miura, Akira Nakamoto, Chikanori Matsumura, Seiryo Sugiura, Toshiaki Hisada

**Affiliations:** Department of Biomedical Engineering, Yamaguchi University, Ube, 755-8611 Japan; Tokuyama Central Hospital, Japan Community Healthcare Organization, Shunan, 745-8522 Japan; The Department of Human and Engineered Environmental Studies, The University of Tokyo, Kashiwa, 277-0871 Japan

**Keywords:** Thorax deformation, Intercostal muscles, Diaphragm, Transversely isotropic hyperelastic material, Finite element method

## Abstract

**Background:**

The biomechanical simulation of the human respiratory system is expected to be a useful tool for the diagnosis and treatment of respiratory diseases. Because the deformation of the thorax significantly influences airflow in the lungs, we focused on simulating the thorax deformation by introducing contraction of the intercostal muscles and diaphragm, which are the main muscles responsible for the thorax deformation during breathing.

**Methods:**

We constructed a finite element model of the thorax, including the rib cage, intercostal muscles, and diaphragm. To reproduce the muscle contractions, we introduced the Hill-type transversely isotropic hyperelastic continuum skeletal muscle model, which allows the intercostal muscles and diaphragm to contract along the direction of the fibres with clinically measurable muscle activation and active force–length relationship. The anatomical fibre orientations of the intercostal muscles and diaphragm were introduced.

**Results:**

Thorax deformation consists of movements of the ribs and diaphragm. By activating muscles, we were able to reproduce the pump-handle and bucket-handle motions for the ribs and the clinically observed motion for the diaphragm. In order to confirm the effectiveness of this approach, we simulated the thorax deformation during normal quiet breathing and compared the results with four-dimensional computed tomography (4D-CT) images for verification.

**Conclusions:**

Thorax deformation can be simulated by modelling the respiratory muscles according to continuum mechanics and by introducing muscle contractions. The reproduction of representative motions of the ribs and diaphragm and the comparison of the thorax deformations during normal quiet breathing with 4D-CT images demonstrated the effectiveness of the proposed approach. This work may provide a platform for establishing a computational mechanics model of the human respiratory system.

## Background

The human respiratory system has a complex structure that stretches from the trachea to the alveoli and involves many physiological phenomena such as the airflow, lung deformation, oxygen exchange, pleural pressure distribution, alveolar pressure distribution, respiratory muscle activation, and chest movement. For such a complicated system, computational mechanics model of the human respiratory system would be a valuable aid to the diagnosis and treatment of these respiratory diseases. For example, chronic obstructive pulmonary disease (COPD) is characterized by the presence of airflow limitation, which is assessed by spirometry test or medical images such as multi detector-row computed tomography (MDCT) [1] in the diagnosis processes. However, the former does not provide information inside of the lung and the later only represents the morphologic feature without the airflow details. Computational models have been proposed to investigate the relationship between imaging measurements and disease severity for developing improved diagnostic methods [2]. On the other hand, computational fluid dynamic analyses have been performed to simulate pharmaceutical aerosol delivery by predicting the site of pharmaceutical aerosol deposition within the airways in the inhaled medicine for treating respiratory diseases [3]. Furthermore, lung deformation simulations have been applied to the radiation therapy for accurate prediction of the lung tumour motion due to breathing [4]. The extreme goal of this research is to build a biomechanical computational model of respiratory system coupling the airflow in the bronchial system with the lung deformation for the diagnosis and treatment of the respiratory disease. To that end, it is first essential to develop an approach to simulating thorax deformation. Because breathing and all subsequent physiological phenomena are closely related to the thorax deformation produced by respiratory muscle contraction, our current goal was to establish a computational mechanics model to simulate the contractions of the diaphragm and intercostal muscles, which are the main muscles responsible for thorax deformation [5].

Thorax deformation consists of the movements of the diaphragm and the rib cage, which mainly result from the contractions of the diaphragm and intercostal muscles during breathing [5]. Previous studies which used the finite element method (FEM) to simulate thorax deformation can be classified into two categories: (1) modelling respiratory muscles with beam elements [6, 7] and (2) representing the rib and diaphragm motions with a prescribed displacement or external load instead of muscle contraction [8–10]. From a biomechanical point of view, a three-dimensional continuum mechanical model of the muscles should be used to simulate the interaction between the muscles and organs inside the chest. Furthermore, physiologically unrealistic displacements and external loads cannot reasonably reproduce the biomechanical environment in the chest. Within the framework of continuum mechanics, a muscle is modelled as an incompressible transversely isotropic hyperelastic material [11] such that the material response in the plane orthogonal to the direction of the muscle fibre is isotropic. However, to date, such material models have not been applied to the formulation of respiratory muscles for simulating thorax deformation.

To overcome the drawbacks faced by previous studies, in this study we set out to simulate the thorax deformation by modelling the respiratory muscles based on continuum mechanics and introducing muscle contractions. First, we constructed a finite element model of the thorax which included the sternum, ribs, vertebrae, intercostal muscles, and diaphragm. To reproduce the muscle contractions based on continuum mechanics, we then introduced a Hill-type transversely isotropic hyperelastic continuum material model which allowed the respiratory muscles to contract along the direction of the fibres with clinically measurable muscle activation. By applying an anatomical muscle fibre orientation and muscle activation to the respiratory muscles, we reproduced physiologically representative motions of the ribs and subsequently the diaphragm. Finally, by activating the inspiratory intercostal muscles and diaphragm simultaneously, we could simulate the thorax deformation during normal breathing, which we compared with four-dimensional computed tomography (4D-CT) images to validate the effectiveness of this approach.

## Methods

### FEM model of thorax

**Fig. 1:**
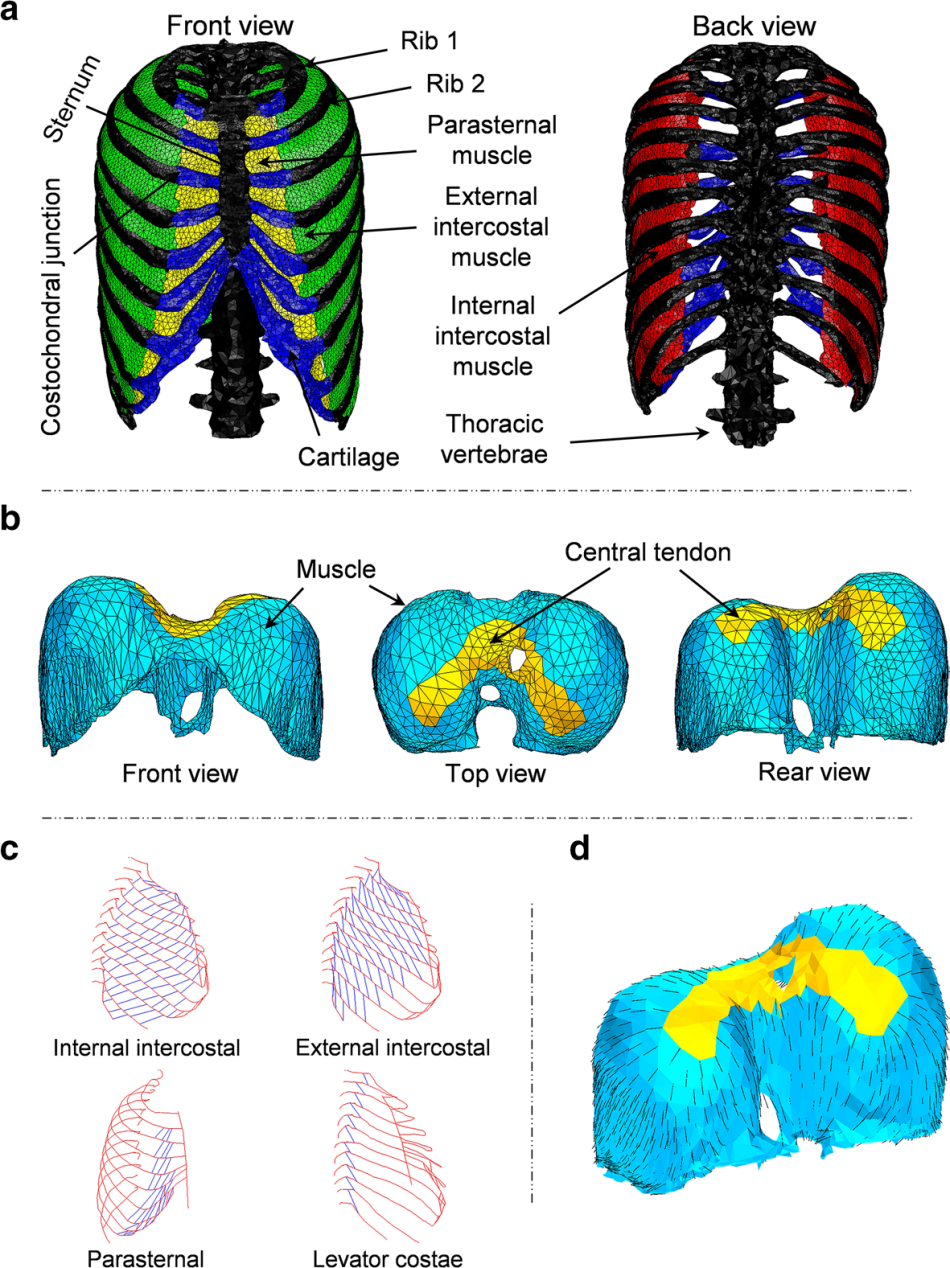
FEM model of the thorax. **a** Respiratory muscles, rib cage, and vertebrae, **b** diaphragm, **c** fibre orientation of the intercostal muscles as measured by Loring (personal communications, 2012), **d** fibre orientation of diaphragm as determined from an anatomy text book [13]

A voxel dataset of the chest was segmented from CT slices of a male volunteer for use in the simulation of the electrophysiological activity of the heart [12]. The CT images were acquired using a CT scanner (Toshiba Aquilion; Toshiba Medical Systems, Japan) with tube voltage 120 kVp tube current of 390 mA. The slice thickness is 0.5 mm and pixel spacing is 0.72 mm for 512 × 512 pixels. This dataset was used to perform a 3D reconstruction of the sternum, ribs, vertebrae, intercostal muscles, and diaphragm which was then rendered into the STL format. Because segmenting the intercostal muscles and diaphragm from the CT slices was difficult, 3D models of the intercostal muscles and diaphragm were constructed in the following way: the positions of intercostal muscles and diaphragm attached to the bones were first determined by referencing an anatomy textbook [13]; then the surfaces of intercostal muscles were created by connecting the muscle attachments between upper and lower rib bones; finally, regarding that diaphragm is the boundary of the chest cavity, the surface of the diaphragm was generated as to attach the lung bottom. The created models of intercostal muscles and diaphragm were confirmed by clinicians. The finite element meshes shown in Fig. 1a, b, consisting of 252,795 tetrahedral elements and 63,057 nodes, were then generated from the 3D models by using the mesh generation software ANSYS ICEM CFD (ANSYS, Inc.). By assuming the intercostal muscles and diaphragm to be incompressible material, the mixed u-p formulation was introduced with three displacement nodes and one hydrostatical pressure node.

Because the diaphragm comes into contact with and slides against the chest wall during breathing [14], contact conditions were applied between the diaphragm and chest wall. By defining the distance between the diaphragm and chest wall as gap function *g* and representing the contact force with *η*, the contact conditions were introduced as $g \ge 0$, $\eta \le 0$, $g \cdot \eta = 0$ by applying Lagrange multiplier method [15]. The first condition ensures that the diaphragm cannot penetrate the chest wall. The second condition means that the contact force has to be compression force. The third condition implies that the contact force can only be generated at where the contact is occurring, i.e. when *g* = 0. All of the degrees of freedom at the bottom of the vertebrae were fixed as a displacement boundary condition. Abdominal pressure and pleural pressure were respectively applied to the lower surface of the diaphragm and inner surface of the thorax with linear variations from 0 to 2 kPa [7] and from −0.5 to −0.75 kPa [5] during normal quiet breathing. The fibre direction of the intercostal muscles was assigned based on human cadaver data obtained from Loring (personal communications, 2012), as shown in Fig. 1c. For the diaphragm, we referenced an anatomy textbook [13] to determine the fibre direction, as shown in Fig. 1d. We assumed that the fibre distribution had a radial pattern from the central tendon to the bottom edge.

### Transversely isotropic hyperelastic material model of respiratory muscles

The intercostal muscles and diaphragm were considered to be incompressible hyperelastic materials. The function $U_{V} = { \det }\varvec{C} - 1$ represents the incompressible constraint and is enforced by the Lagrange multiplier $\xi$, where ${ \det }\varvec{C}$ is the determinant of the right Cauchy-Green tensor $\varvec{C}$. To exclude the effect of the volume-changing deformation, the strain energy $U_{R}$ was assumed to depend on the first reduced invariant of $\varvec{C}$. This was defined as $\overline{I}_{1}^{C} = J^{ - 2/3} {\text{tr}}\varvec{C}$, where $J = { \det }\varvec{F}$ denotes the determinant of the deformation gradient $\varvec{F}$. By denoting ***t*** as the external conservative force on the Neumann boundary, the total potential energy $\Phi$ of the muscles can be formulated as a function of the displacement ***u*** and Lagrange multiplier $\xi$, as shown in Eq. (1):

1 $$\Phi \left( {\varvec{u},\xi } \right) = \int_{\varOmega } {U_{R} {\text{d}}\varOmega } + \int_{\varOmega } {\xi U_{V} {\text{d}}\varOmega } - \int_{\partial \varOmega } {\varvec{t} \cdot \varvec{u}{\text{dS}}} .$$

Given the principle of stationary potential energy, the variation in $\Phi$ is equal to zero:

2 $$\delta \Phi = \int_{\varOmega } {\frac{{\partial U_{R} }}{{\partial \varvec{E}}}\delta \varvec{E}{\text{d}}\varOmega } + \int_{\varOmega } {\left( {\xi \frac{{\partial U_{V} }}{{\partial \varvec{E}}}\delta \varvec{E} + \delta \xi U_{V} } \right)} {\text{d}}\varOmega - \int_{\partial \varOmega } {\varvec{t} \cdot \delta \varvec{u}{\text{dS}}} = 0,$$

where ${\mathbf{E}}$ is the Green–Lagrange strain tensor. Thus, the weak form of the governing equation is as follows:

3 $$\left\{ \begin{array}{l} \int_{\varOmega } {\delta \varvec{E}\left( {\frac{{\partial U_{R} }}{{\partial \varvec{E}}} + \xi \frac{{\partial U_{V} }}{{\partial \varvec{E}}}} \right){\text{d}}\varOmega } - \int_{\partial \varOmega } {\varvec{t} \cdot \delta \varvec{u}{\text{dS}}} = 0 \hfill \\ \int_{\varOmega } {\delta \xi U_{V} {\text{d}}\varOmega } = 0 \hfill \\ \end{array} \right..$$

**Fig. 2:**
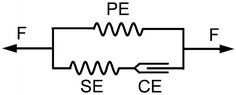
Hill’s three-element muscle model

The Lagrange multiplier corresponds to the hydrostatic pressure $p$ by $p = - 2\xi$ [16]. In this study, the strain energy function $U_{R}$ proposed by Martins et al. [11] was introduced to model the skeletal muscles. This model extends the traditional Hill’s three-element muscle model to three dimensions. As shown in Fig. 2, the parallel element (PE) and series element (SE) are nonlinear springs which represent passive behaviour. The contractile element (CE) produces a contractile force when the muscle is excited.

The muscle force $F$ can be assumed to be the sum of the forces in the PE and CE or SE:

4 $$F = F^{\text{PE}} + F^{\text{SE}} = F^{\text{PE}} + F^{\text{CE}} .$$

Then, the nominal stress along the fibre direction $T$ can be written as:

5 $$T = T^{\text{PE}} + T^{\text{SE}} = T^{\text{PE}} + T^{\text{CE}} .$$

**Fig. 3:**
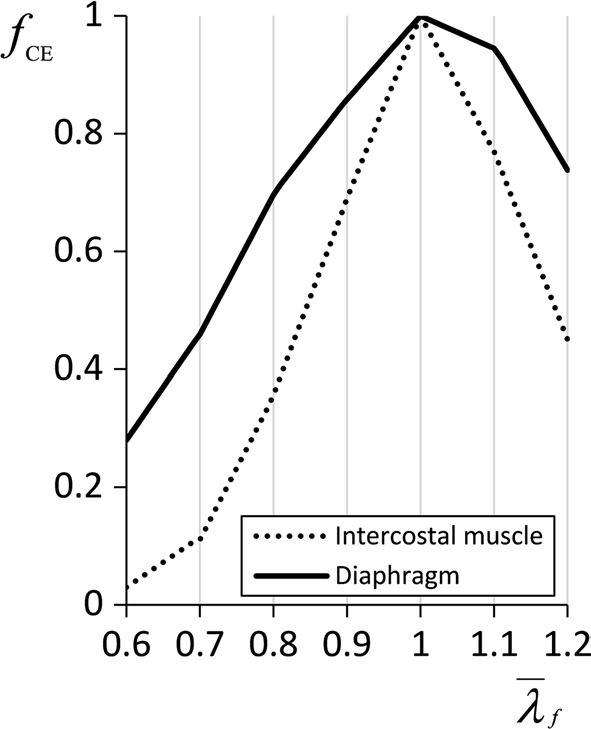
Active force–length relationship $f_{\text{CE}} \left( {\overline{\lambda }_{f} } \right)$ of the diaphragm and intercostal muscles of an adult baboon. The *solid line* represents the diaphragm; the *dotted line* represents the intercostal muscle

The stress $T^{\text{PE}}$ is defined as follows:

6 $$T^{\text{PE}} = T_{0}^{\text{M}} f_{\text{PE}} \left( {\overline{\lambda }_{f} } \right)$$

7 $$f_{\text{PE}} \left( {\overline{\lambda }_{f} } \right) = \left\{ \begin{aligned} 2aA\left( {\overline{\lambda }_{f} - 1} \right)e^{{a\left( {\overline{\lambda }_{f} - 1} \right)^{2} }} ,\quad {\text{for }}\overline{\lambda }_{f} > 1 \hfill \\ 0 ,\quad \quad \quad \quad \quad \quad \quad \quad {\text{otherwise}} \hfill \\ \end{aligned} \right.,$$

where $\overline{\lambda }_{f}$ is the stretch ratio in the fibre direction given by $\overline{\lambda }_{f} = \left[ {J^{ - 2/3} \varvec{C}:\left( {\varvec{N} \otimes \varvec{N}} \right)} \right]^{1/2}$. ***N*** is the initial direction of the muscle fibre, $T_{0}^{\text{M}}$ denotes the maximum muscle nominal stress determining the maximum muscle contraction force, *a* and *A* are material constants [11], and $f_{\text{PE}} \left( {\overline{\lambda }_{f} } \right)$ is the passive force–length relationship of the parallel element PE [11]. The stress $T^{\text{CE}}$ is given by

8 $$T^{\text{CE}} = T_{0}^{\text{M}} f_{\text{CE}} \left( {\overline{\lambda }_{f} } \right)\alpha \left( {\text{t}} \right)\gamma ,$$

where *γ* represents the muscle’s activation level over a range from 0 to 1, *α* represents the time dependence of the activation and for which the maximum value can be 1, and $f_{\text{CE}} \left( {\overline{\lambda }_{f} } \right)$ is the active force–length relationship of the contractile element CE. In this study, this was considered to be the active force–length relationship for the muscle tissue. Figure 3 shows the experimental data for the active force–length relationship of the intercostal muscles and diaphragm as measured from an adult baboon [17].

According to the experimental data, $f_{\text{CE}} \left( {\overline{\lambda }_{f} } \right)$ of the intercostal muscles can be formulated as

9 $$f_{\text{CE}}^{Int} \left( {\overline{\lambda }_{f} } \right) = \left\{ \begin{array}{ll} 0.85\overline{\lambda }_{f} - 0.48, &\quad {\text{for }}0.6 \le \overline{\lambda }_{f} < 0.7 \hfill \\ 2.43\overline{\lambda }_{f} - 1.59, &\quad {\text{for }}0.7 \le \overline{\lambda }_{f} < 0.8 \hfill \\ 3.32\overline{\lambda }_{f} - 2.30, &\quad {\text{for }}0.8 \le \overline{\lambda }_{f} < 0.9 \hfill \\ 3.10\overline{\lambda }_{f} - 2.10, &\quad {\text{for }}0.9 \le \overline{\lambda }_{f} < 1.0 \hfill \\ - 2.30\overline{\lambda }_{f} + 3.30, &\quad {\text{for 1}} .0\le \overline{\lambda }_{f} < 1.1 \hfill \\ - 3.20\overline{\lambda }_{f} + 4.29, &\quad {\text{for 1}} .1\le \overline{\lambda }_{f} < 1.2 \hfill \\ 0, &\quad{\text{otherwise}} \hfill \\ \end{array} \right..$$

Then, $f_{\text{CE}} \left( {\overline{\lambda }_{f} } \right)$ of the diaphragm can be formulated as

10 $$f_{{{\text{CE}}}}^{{Dia}} \left( {\overline{\lambda }_{f} } \right) = \left\{ {\begin{array}{*{20}l} {1.80\overline{\lambda }_{f} - 0.80,} & {\quad {\text{for }}0.6 \le \overline{\lambda }_{f} < 0.7} \hfill \\ {2.37\overline{\lambda }_{f} - 1.20,} & {\quad {\text{for }}0.7 \le \overline{\lambda }_{f} < 0.8} \hfill \\ {1.61\overline{\lambda }_{f} - 0.59,} & {\quad {\text{for }}0.8 \le \overline{\lambda }_{f} < 0.9} \hfill \\ {1.42\overline{\lambda }_{f} - 0.42,} & {\quad {\text{for }}0.9 \le \overline{\lambda }_{f} < 1.0} \hfill \\ { - 0.55\overline{\lambda }_{f} + 1.55,} & {\quad {\text{for 1}}.0 \le \overline{\lambda }_{f} < 1.1} \hfill \\ { - 2.11\overline{\lambda }_{f} + 3.27,} & {\quad {\text{for }}1.1 \le \overline{\lambda }_{f} < 1.2} \hfill \\ {0,} & {\quad {\text{otherwise}}} \hfill \\ \end{array} } \right..$$

**Fig. 4:**
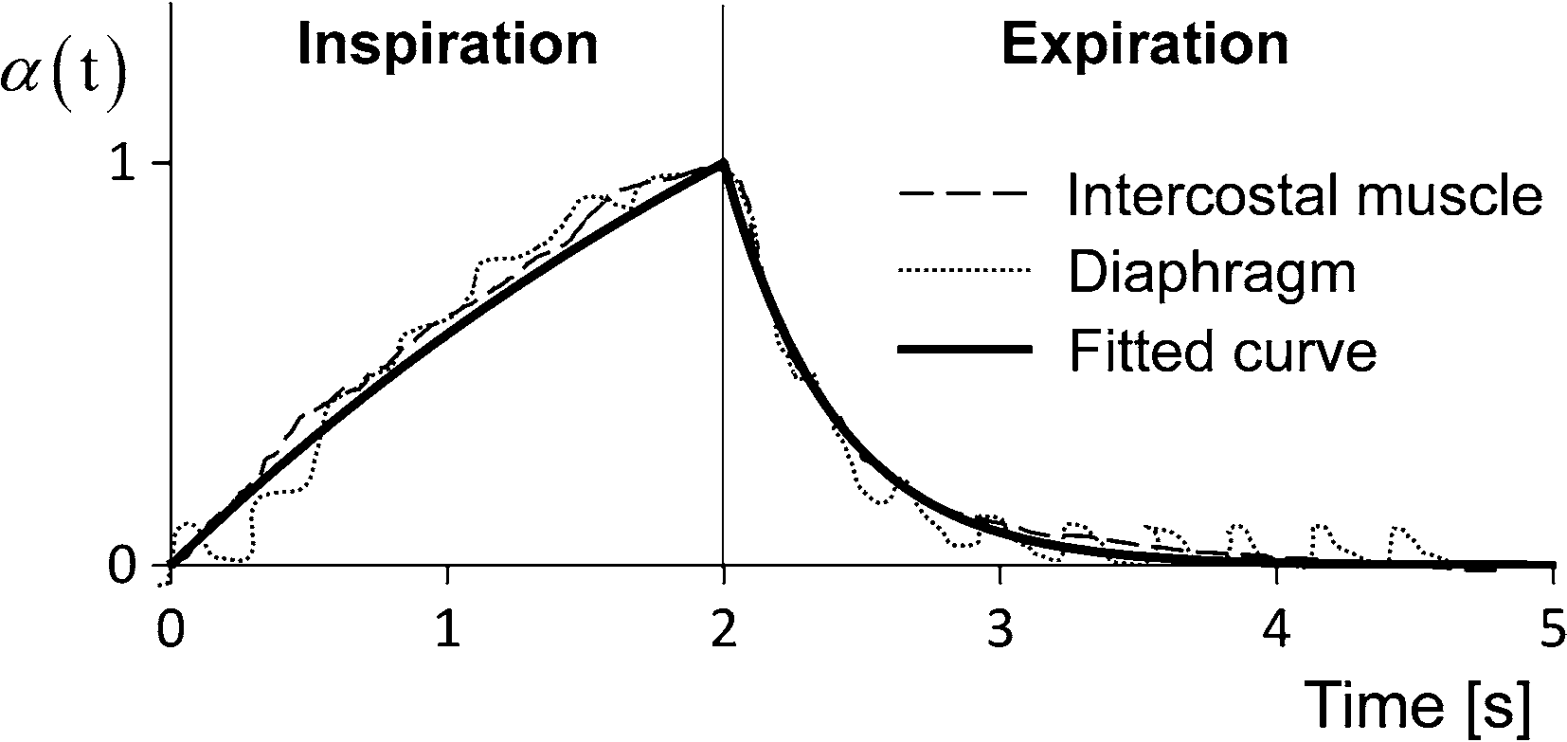
Time dependence of activation $\alpha \left( {\text{t}} \right)$ of the diaphragm and intercostal muscles

The time dependence of the activation $\alpha \left( {\text{t}} \right)$ of the intercostal muscles and diaphragm was fitted to experimental data obtained from dogs [18], as shown in Fig. 4. Concerning the parameters $f_{\text{CE}} \left( {\overline{\lambda }_{f} } \right)$ and $\alpha \left( {\text{t}} \right)$, by considering that the biological characteristics are similar for mammalia and the expected difference between species can be represented by the different magnitudes, in this study, the normalized $f_{\text{CE}} \left( {\overline{\lambda }_{f} } \right)$ and $\alpha \left( {\text{t}} \right)$ from animal experiment shown in Figs. 3 and 4 were introduced and their magnitudes were then adjusted to be able to reproduce the behaviour of human.

The muscles were modelled as incompressible transversely isotropic hyperelastic materials for which the strain energy function can be written as follows:

11 $$U_{R} = U_{I} \left( {\overline{I}_{1}^{C} } \right) + U_{f} \left( {\overline{\lambda }_{f} ,\alpha ,\gamma } \right)$$

12 $$U_{I} = ce^{{b\left( {\overline{I}_{1}^{C} - 3} \right) - 1}}$$

13 $$U_{f} \left( {\overline{\lambda }_{f} ,\alpha ,\gamma } \right) = U_{\text{PE}} \left( {\overline{\lambda }_{f} } \right) + U_{\text{CE}} \left( {\overline{\lambda }_{f} ,\alpha ,\gamma } \right)$$

14 $$U_{\text{PE}} \left( {\overline{\lambda }_{f} } \right) = T_{0}^{\text{M}} \int_{1}^{{\overline{\lambda }_{f} }} {f_{\text{PE}} \left( \lambda \right){\text{d}}\lambda }$$

15 $$U_{\text{CE}} \left( {\overline{\lambda }_{f} ,\alpha ,\gamma } \right) = T_{0}^{\text{M}} \int_{1}^{{\overline{\lambda }_{f} }} {f_{\text{CE}} \left( \lambda \right)\alpha \left( {\text{t}} \right)\gamma {\text{d}}\lambda } ,$$

where $U_{I}$ and $U_{f}$ are the strain energies stored in the isotropic hyperelastic matrix and muscle fibre, respectively, and $c$ and $b$ are material constants. For an incompressible hyperelastic material, by denoting $p$ as the hydrostatic pressure, the second Piola–Kirchhoff stress tensor ***S*** can be derived as follows:

16 $$\begin{aligned} \varvec{S} & = - p\varvec{C}^{ - 1} + \frac{{\partial U_{R} }}{\partial E} = - p\varvec{C}^{ - 1} + \frac{{\partial U_{I} }}{{\partial \overline{I}_{1}^{C} }}\frac{{\partial \overline{I}_{1}^{C} }}{{\partial \varvec{E}}} + \frac{{\partial U_{f} }}{{\partial \overline{\lambda }_{f} }}\frac{{\partial \overline{\lambda }_{f} }}{{\partial \varvec{E}}} \\ & = - p\varvec{C}^{ - 1} + \left\{ {bce^{{b\left( {\overline{I}_{1}^{C} - 3} \right)}} } \right\}\left( {2\varvec{I} - \frac{2}{3}\overline{I}_{1}^{C} \varvec{C}^{ - 1} } \right) \\ &\quad + \left\{ {T_{0}^{M} \left( {f_{PE} \left( {\overline{\lambda }_{f} } \right) + f_{CE} \left( {\overline{\lambda }_{f} } \right)\alpha \left( t \right)\gamma } \right)} \right\}\left( {\overline{\lambda }_{f}^{ - 1} \left( {\varvec{N} \otimes \varvec{N}} \right) - \frac{1}{3}\overline{\lambda }_{f} \varvec{C}^{ - 1} } \right). \\ \end{aligned}$$

The material parameters used for the intercostal muscles and the diaphragm were $T_{0}^{\text{M}}$ = 0.3 MPa [17], *c* = 0.009348 MPa, and *b* = 1.4939 MPa [19]. The Lamé parameters *μ* and *λ* of the isotropic elastic model were 569.23 and 853.85 MPa, respectively for bone [6]; 65.38 and 98.08 MPa, respectively, for cartilage [6]; and 5.18 and 10.05 MPa, respectively, for tendons [20].

### Simulation procedure

In this study, by omitting the inertial force, the static analyses were performed to examine and validate the introduced Hill-type transversely isotropic hyperelastic continuum skeletal muscle model for thorax deformation. The muscle contraction was activated by assigning parameter *γ* in Eq. (8) to be greater than zero. First, we individually activated the intercostal muscles and diaphragm to separately investigate the effect of each muscle on the chest deformation, and also to confirm the representative motions of the ribs and the diaphragm. Then, we simultaneously activated the intercostal muscles and diaphragm in order to simulate the thorax deformation. Finally, the resultant thorax deformation was compared with 4D-CT data during normal quiet breathing. The 4D-CT images of a healthy adult male were acquired using a 320-MDCT scanner (Aquilion One, Toshiba Medical Systems, Japan) for three phases during inspiration and expiration, respectively. To reduce radiation exposure, the detector row was reduced to 160, the tube voltage was reduced to 80 kVp and the tube current was reduced to 30 mA. The field of view was set to 16 cm to cover the diaphragm. The data were collected at Tokuyama Central Hospital, Japan Community Healthcare Organization, based on the Declaration of Helsinki. The procedure was approved by the Ethics Committee of the hospital.

## Results

Based on the formulation given in the section entitled ‘Transversely isotropic hyperelastic material model of respiratory muscle’, the incompressible transversely isotropic hyperelastic material model was implemented in a nonlinear finite element analysis program developed in-house, which was validated in the authors’ previous works. The thorax deformation was simulated by using the FEM model presented in the section entitled ‘FEM model of thorax’. The average simulation time was 630 min with 3.5 GHz Intel Core i7 and 32 GB RAM, and the actual computational time (CPU time) occupied approximately 90 % of the simulation time.

### Rib motion

The intercostal muscles were activated based on the muscle activity distribution during inspiration and expiration to obtain the corresponding chest movements [21, 22]. The parasternal intercostal muscles and external intercostal muscles were activated for the inspiratory chest movement, while the internal intercostal muscles were activated to reproduce the expiratory chest movement. For the parasternal muscles, according to the measured muscle activation from nine adult mongrel dogs with weight ranging from 14 to 21 kg [21], the muscle activation reached a maximum near the sternum (*γ* = 1.0) but decreased to zero in the lateral direction at the costochondral junctions. According to the measured muscle activation from eight adult mongrel dogs with weight ranging from 12 to 29 kg [22], the largest muscle activation among the external intercostal muscles occurred in the dorsal region of the interspace between ribs 1 and 2 (*γ* = 1.0) and gradually decreased in the caudal and ventral directions through to the lower ribs. For the internal intercostal muscles, also according to the Ref. [22], we activated the muscles in the lowest rib interspace with the largest activation (*γ* = 1.0), while the activation gradually decreased in the cranial and ventral directions through to the upper ribs.

**Fig. 5:**
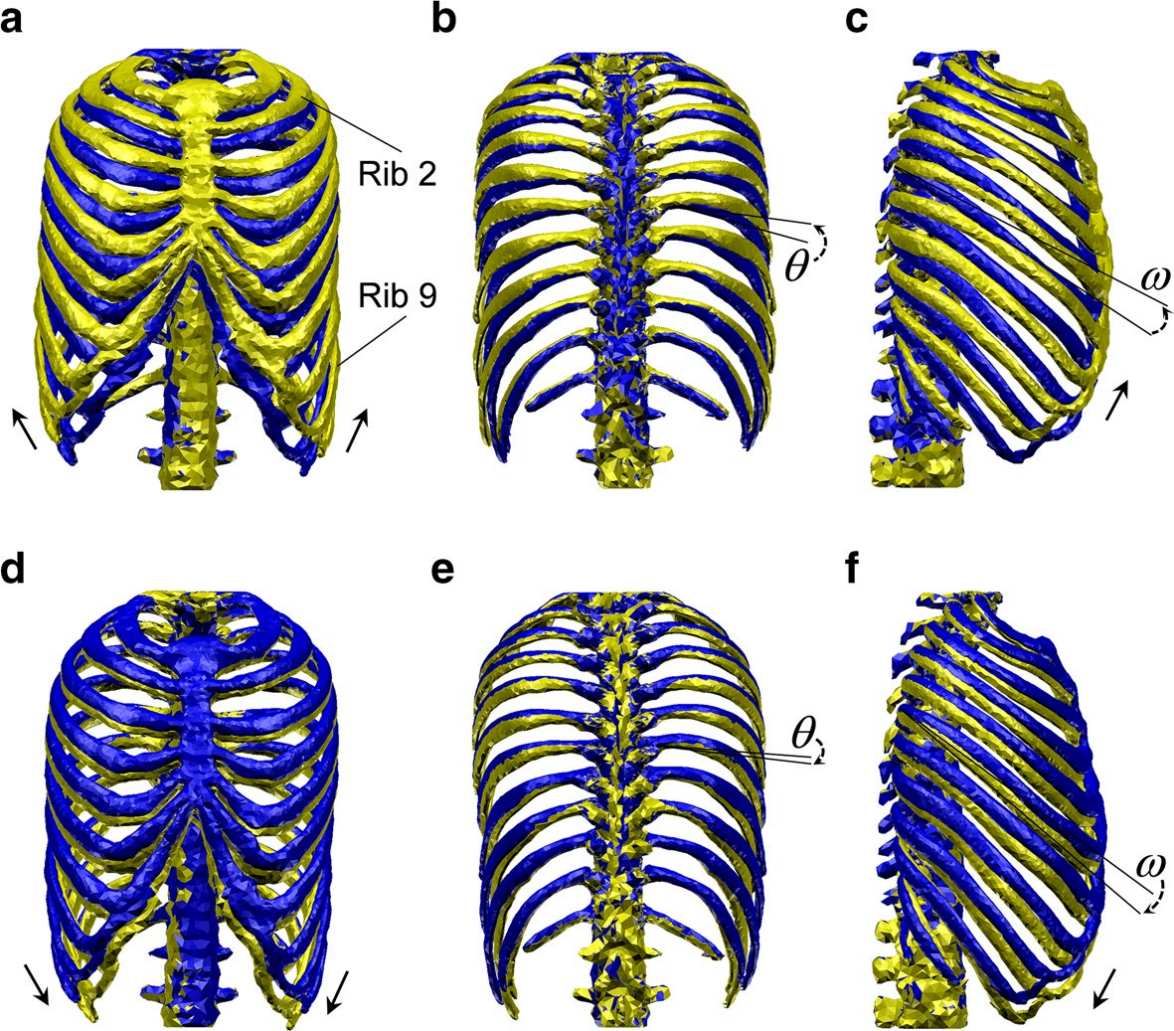
Rib motion generated by the intercostal muscles. The *golden shape* represents the state after muscle contraction; the *blue shape* represents the state before muscle contraction. **a**–**c** illustrate the inspiratory chest movement, and **d**–**f** illustrate the expiratory chest movement. **a**, **d** Front view, **b**, **e** rear view, **c**, **f** lateral view

As a result, we obtained the inspiratory (Fig. 5a–c) and expiratory (Fig. 5d–f) movements of the rib cage. During inspiration, the front (Fig. 5a) and rear (Fig. 5b) views of the rib cage showed that the transverse diameter increased, especially for the lower ribs. The lateral view (Fig. 5c) showed the extension of the anteroposterior diameter. These increases in the chest diameter demonstrated that the obtained chest movements were inspiratory. For the expiratory chest movements, the front views (Fig. 5d, e) showed that the rib cage narrowed medially (i.e. transverse diameter decreased), particularly in the case of the lower ribs. The lateral view (Fig. 5f) showed the decrease in the anteroposterior diameter. These decreases in the chest diameter demonstrated that the obtained chest movements were expiratory.

More importantly, among the inspiratory (Fig. 5a–c) and expiratory (Fig. 5d–f) chest movements, we observed representative rib motions which have been characterized physiologically. The arrows in Fig. 5a, d show the directions of the rib movements. The ribs moved like the handle of a bucket, which is known as the ‘bucket-handle motion’ in physiology. The arrows in Fig. 5c, f show that the ribs moved like the handle of an old-fashioned water pump, which is the source of the term ‘pump-handle motion’. The angle *θ* shown in Fig. 5b, e is thus called the ‘bucket-handle angle’ of the rib, and the angle *ω* shown in Fig. 5c, f is called the ‘pump-handle angle’ of the rib, as explained by Troyer et al. [23]. The angles *θ* and *ω* were measured for ribs 2–9 in a lung inflation experiment and were found to be equivalent to the inspiratory muscle contraction studied by Troyer et al. [23].

**Fig. 6:**
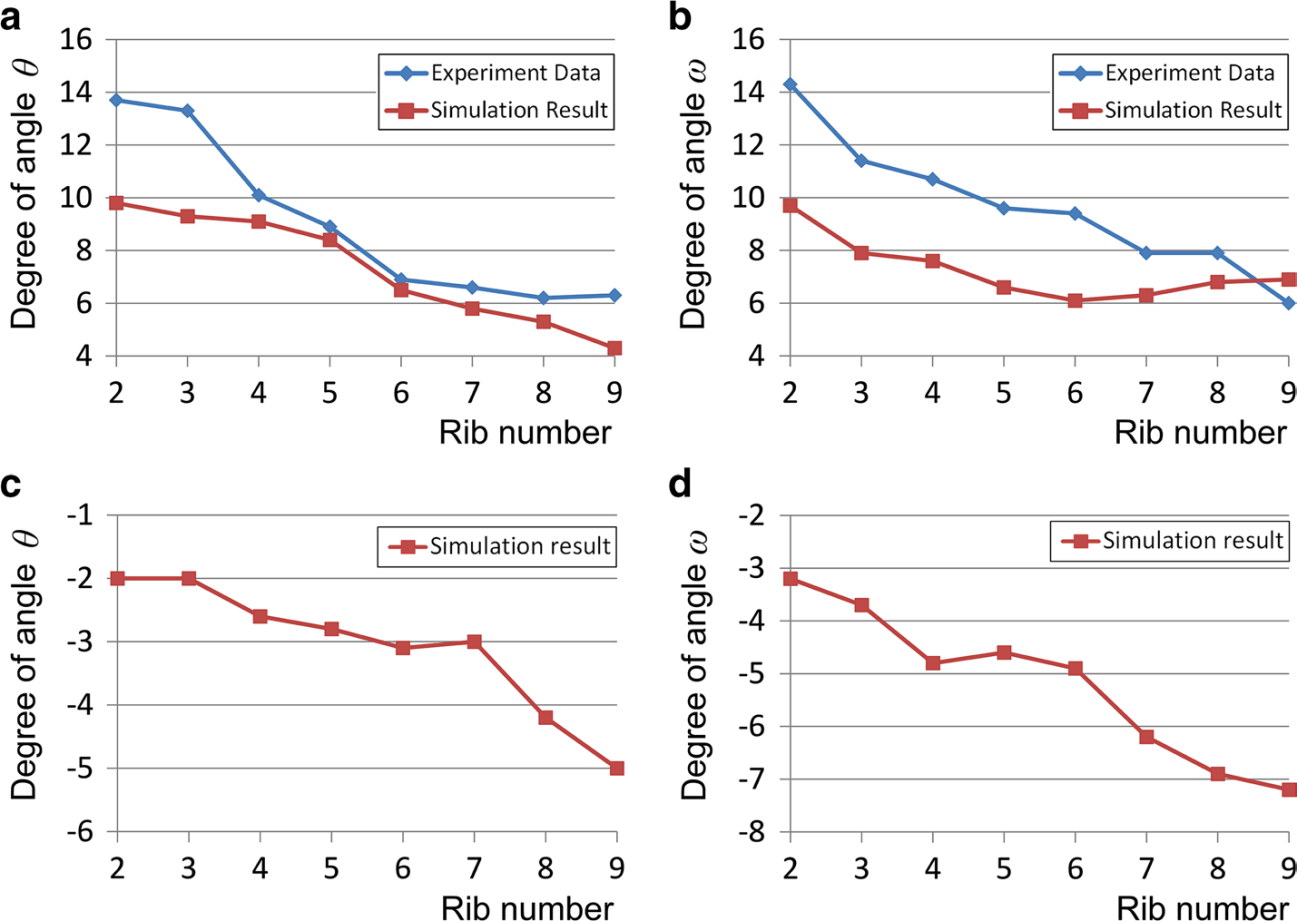
Bucket-handle angle *θ* and pump-handle angle *ω* of ribs 2–9. The *horizontal axis* shows the rib number, and the *vertical axi*s shows angles *θ* and *ω*. **a** Comparison of simulated and experimental [23] bucket-handle angle *θ* during inspiratory chest movement, **b** comparison of simulated and experimental [23] pump-handle angle *ω* during inspiratory chest movement, **c** simulated bucket-handle angle *θ* during expiratory chest movement, **d** simulated pump-handle angle *ω* during expiratory chest movement

To confirm the role of the intercostal muscles in the reproduction of the bucket-handle and pump-handle motions and to validate our approach, we compared the simulated angles *θ* and *ω* of ribs 2–9 generated by the inspiratory intercostal muscles with the experimental measurements [23], as shown in Fig. 6a, b. The horizontal axis shows the rib number, and the vertical axis shows the angles *θ* and *ω*. We observed that the simulation results were similar to the experimental measurements: the upper ribs rotated more than the lower ribs for both the bucket-handle (angle *θ*) and pump-handle (angle *ω*) motions. The deviation in the bucket-handle angle *θ* appeared at the upper ribs, which rotated more in the experimental data compared with the simulation results. For most of the ribs, the pump-handle angle *ω* was smaller than those in the experimental data. Figure 6c, d also summarize angles *θ* and *ω* generated by the expiratory intercostal muscles. The anticlockwise direction is taken as positive; the upper ribs rotated less than the lower ribs.

### Diaphragm motion

**Fig. 7:**
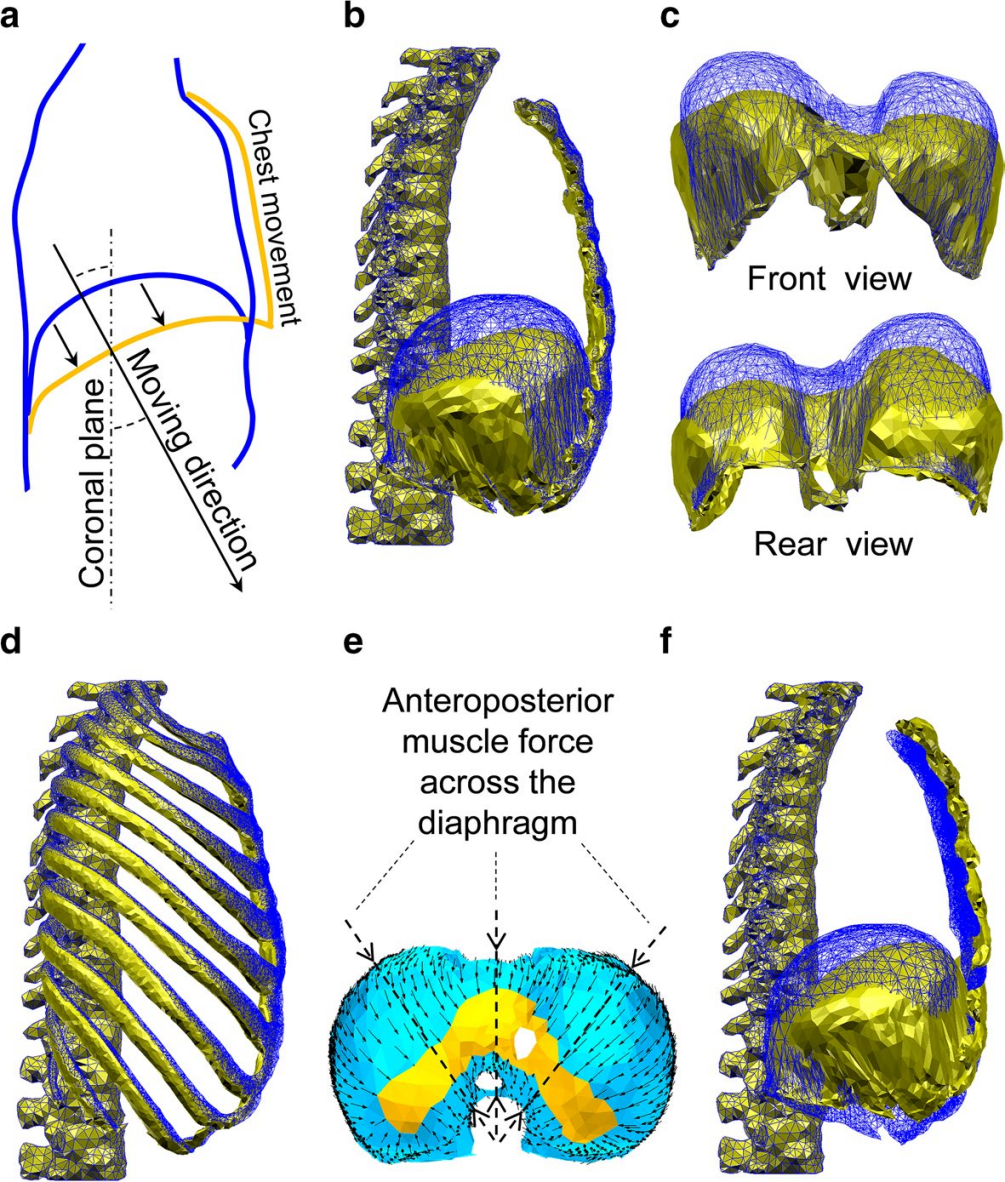
Diaphragm motion. The *golden shape* represents the state after muscle contraction; the *blue shape* represents the state before muscle contraction. **a** Clinical observation of the diaphragm motion and chest movement on the sagittal plane in MRI scans [24–26], **b** simulation results of the right hemidiaphragm for comparison with the clinical observation, **c** *front* and *rear view* of the diaphragm deformation, **d** motion of the rib cage caused by isolated contraction of the diaphragm (amplified ×2), **e** distribution of muscle forces based on the radial fibre orientation in the diaphragm, **f** simulation results of the right hemidiaphragm from simultaneous activation of the diaphragm and inspiratory intercostal muscles for comparison with the clinical observation

In order to confirm the simulation performance of the diaphragm motion, we first simulated the isolated contraction of the diaphragm (*γ* = 1.0) without the activation of the other inspiratory muscles in the FEM chest model. Figure 7a shows a clinical observation of the sagittal plane of the diaphragm dome based on MRI scans from 25 healthy subjects (10 females and 15 males) with mean age 31.9 years; average weight 72.5 kg; average height 174.1 cm [24–26], which not only descended caudally but also exhibited some movement in the ventral direction accompanied by inspiratory chest movement during breathing. Figure 7b illustrates a lateral view of the simulated diaphragm deformation for comparison with the clinical observation. The dome of the diaphragm exhibited a similar movement to the clinical observation shown in Fig. 7a. The front and rear views in Fig. 7c show that the diaphragm dome also descended caudally. However, regarding the ribs and sternum, contrary to the MRI scans, the isolated diaphragm contraction generated an expiratory rib rotation whereby the ribs and sternum were simultaneously pulled dorsally and caudally, as shown in Fig. 7d (the deformation is amplified twice for visualization). Such a movement may have resulted from the anteroposterior muscle force acting on the top of the diaphragm and was consistent with the fibre direction of the diaphragm shown in Fig. 7e. The collaboration between the intercostal muscles and diaphragm in the thorax deformation was confirmed when the intercostal muscles and diaphragm were contracted simultaneously. In this case, a thorax deformation consistent with the MRI scans was obtained in the simulation, as shown in Fig. 7f.

### Simulating the thorax deformation during normal quiet breathing and comparison with 4D-CT images

After confirming the performance of the intercostal muscles and diaphragm, we simultaneously activated the external intercostal muscles, parasternal intercostal muscles, and diaphragm to simulate the thorax deformation during normal quiet breathing. For the external intercostal and parasternal intercostal muscles, the same activation distribution as that described in the section entitled ‘Rib motion’ was adopted, and the activation level *γ* was set to 0.3 for the inspiratory intercostal muscles and diaphragm based on the results of experiments with spontaneously breathing dogs [27]. The simulated deformation results were compared with the 4D-CT images captured during normal quiet breathing in order to validate our findings.

**Fig. 8:**
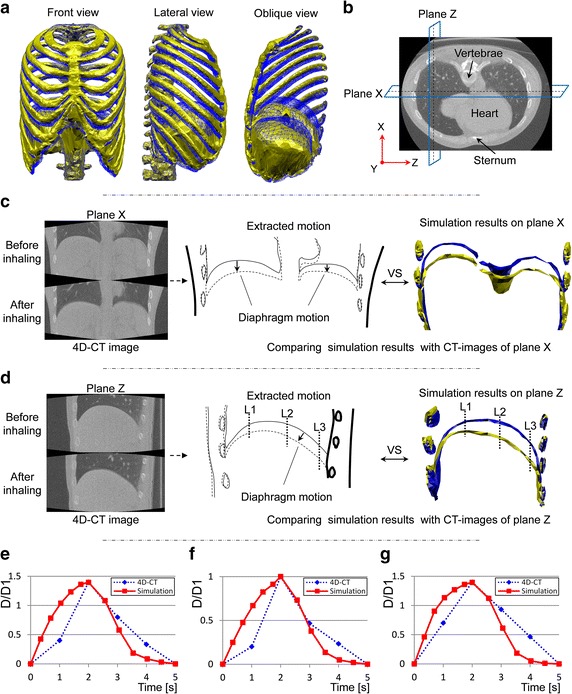
Simulation results and observation of CT images during normal quiet breathing. **a** Deformation of thorax, **b** location of *planes X* and *Z* in the chest, **c** CT observation of *plane X* (the *dashed line* represents the state after inhaling; the *solid line* represents the state before inhaling) compared with the simulation results (the *golden shape* represents the state after muscle contraction; the *blue shape* represents the state before muscle contraction), **d** CT observation of *plane Z* (the *dashed line* represents the state after inhaling; the *solid line* represents the state before inhaling) compared with the simulation results (the *golden shape* represents the state after muscle contraction; the *blue shape* represents the state before muscle contraction), **e** movement at location L1 on plane Z compared with 4D-CT images, **f** movement at location L2 on *plane Z* compared with 4D-CT images, **g** movement at location L3 on *plane Z* compared with 4D-CT images

Figure 8a shows the resultant thorax deformation. The front and lateral views show that the transverse and anteroposterior diameters of the thorax were slightly increased by the bucket-handle and pump-handle motions of ribs, respectively. The oblique view illustrates the diaphragm deformation inside the chest. For the movements of the diaphragm observed with CT images, we chose planes X and Z, (shown in Fig. 8b) which did not cross the heart, and extracted the movements of the ribs and the diaphragm from the 4D-CT images, as shown in Fig. 8c, d. The solid line represents the state before inhalation, while the dashed line corresponds to the state after inhalation. The arrows illustrate the movement direction of the diaphragm tops for visualization. The arrows in Fig. 8c show that the diaphragm moved in the craniocaudal direction. The arrow in Fig. 8d illustrates the oblique moving direction of the diaphragm top, which not only descended in the craniocaudal direction but also in the dorsal–ventral direction. Correspondingly, the simulated deformations shown on the right-hand side of Fig. 8c, d represent the same motion observed in the CT images. The dashed lines in Fig. 8c, d show the rib movements extracted from the CT images. Even though the inspiratory rib movement was small during normal quiet breathing, the simulation results were consistent with the CT images: the ribs slightly rotated cranially, laterally and ventrally, and the circumference of the chest increased, which represented the inspiratory rib motion. We also compared the time variation of the diaphragm deformation with the 4D-CT images. Figure 8d shows the three locations on plane Z chosen for comparison, which are marked with dotted lines and labelled L1, L2, and L3. Figure 8e–g show the compared results. The vertical axis (D/D1) in Fig. 8e–g shows the displacement normalized against that of location L1, i.e. D is the displacement of each location and D1 is the displacement of location L1. The horizontal axis corresponds to the elapsed time during quiet breathing from 0 to 5 s. The dotted line corresponds to the 4D-CT images, while the solid line represents the simulation results.

## Discussion

To simulate the thorax deformation, we modelled the respiratory muscles based on continuum mechanics while considering muscle contractions. The introduction of the Hill-type transversely isotropic hyperelastic continuum skeletal muscle model allowed the respiratory muscles to contract along the direction of the fibres and thus reproduced the representative motions of the ribs and diaphragm according to physiology. In this study, we focused on validating the effectiveness of this approach at simulating the thorax deformation driven by the diaphragm and the intercostal muscles, which are significant for human breathing.

### Rib motion

Resultant chest movement demonstrated the importance of the intercostal muscles to breathing because they can expand (Fig. 5a–c) and contract (Fig. 5d–f) the rib cage. Besides the changes in the diameter of the rib cage, we were also able to reproduce the representative bucket-handle and pump-handle motions of the ribs. In the same manner as described in the literature [28], the bucket-handle motion primarily appeared in the lower ribs, for which the transverse diameter changes (Fig. 5a, d) were larger than the changes in the anteroposterior diameter (Fig. 5c, d). For the upper ribs, the changes in the anteroposterior diameter of the upper ribs prominently expressed the pump-handle motion (Fig. 5). Hence, the intercostal muscles were found to have the primary function of producing the bucket-handle and pump-handle motions during human breathing.

In addition, as shown in Fig. 6a, b, the rib rotation angles *θ* and *ω* for bucket-handle and pump-handle motions were close to the experimental measurements [23]. This demonstrated that our approach of activating the inspiratory intercostal muscles successfully reproduced the bucket-handle and pump-handle motions for each rib, which actually occur during human inspiration. Consequently, the obtained inspiratory and expiratory chest movements and reproduction of the representative rib motions validated our approach of simulating rib motions driven by the inspiratory intercostal muscles.

Regarding the deviation of the upper ribs, which rotated more in the experimental data than in the simulation results, we believe that this was because we did not consider the other inspiratory muscles which also contract during human deep breathing, especially the cervical accessory muscles which act on top of the rib cage. Although the external intercostal muscles are the most important muscles for elevating the rib cage, the cervical accessory muscles (i.e. sternocleidomastoid and scalene muscles) also help elevate the upper ribs [5]. Loring’s FEM analysis demonstrated that the cervical accessory muscles elevate the entire rib cage and especially cause the pump-handle motion for each rib [6]. Regarding the simulation results, Fig. 6a, b show that the deviation of the bucket-handle angle was relatively large in the upper ribs, whereas the pump-handle angles were smaller than the experimental measurements for each rib. These results imply that the cervical accessory muscles may have a greater influence on elevating the rib cage during deep breathing.

Although we are unaware of any experimental data for rib rotation during expiration, because the internal intercostal muscles in the caudal–dorsal area exhibited the greatest activity during expiration [22], the results of our simulation showed that the internal intercostal muscles could rotate the lower ribs more than the upper ribs during expiration, as shown in Fig. 6c, d.

Consequently, this examination demonstrated that the Hill-type transversely isotropic hyperelastic continuum skeletal muscle model provides an effective means of simulating rib motions driven by the intercostal muscles.

### Diaphragm motion

For the simulation of the diaphragm motion, we first confirmed the ability of the proposed approach to reproducing the representative motion of the diaphragm as observed clinically by MRI scans. In these clinical observations, the diaphragm moves in the craniocaudal direction at an angle to the coronal plane, as shown in Fig. 7a. This movement characterizes the fact that the diaphragm not only moves vertically like a piston but also moves in the caudal-ventral direction. In the simulation with isolated diaphragm contractions, as shown in Fig. 7b, the dome of the diaphragm on the sagittal plane moved in the same directions (caudally and ventrally) as those observed with MRI scans. The front and rear views in Fig. 7c show that the diaphragm moved vertically like a piston. This diaphragm motion can be attributed to its anatomical structure, in that the muscle fibres are short in the area between the sternum and anterior tendon while the longer muscles are in the posterior part and on both sides of the diaphragm (Fig. 1b). Although the muscles in the anterior part of the diaphragm also lower the tendon, the displacement is smaller than in the other areas. Therefore, the diaphragm moved in the craniocaudal direction at an angle to the coronal plane (Fig. 7b, c).

Note that the isolated contraction of the diaphragm pulled the ribs dorsally and caudally to result in an expiratory rib motion (Fig. 7d) due to the anteroposterior muscle force (Fig. 7e) on top of the diaphragm dome. Clearly, such deformation differs from the clinical observation shown in Fig. 7a, where the chest movements are inspiratory. To investigate the reason for the deviation, we simultaneously activated the inspiratory intercostal muscles and the diaphragm, and the resultant deformation is shown in Fig. 7f. The comparison between Fig. 7a, f clearly shows that an inspiratory chest movement and diaphragm motion similar to the clinical observation were obtained in the simulation.

Note that the effects of the diaphragm and inspiratory intercostal muscles were opposite because the contraction of the diaphragm could caudally and dorsally rotate the ribs to extend the inspiratory intercostal muscle fibres, whereas the contraction of the inspiratory intercostal muscles could cranially and ventrally rotate the ribs to lengthen the diaphragm muscle fibres. This phenomenon is consistent with the experimental observation by DiMarco et al. [29]. The significance of this phenomenon is primarily related to the active force–length relationship (Fig. 3). The active force–length relationship determines the optimal length for exerting muscle force. Thus, during the contraction of the diaphragm, the length of the contracted muscle fibres gradually diverges from the optimal length. If the inspiratory intercostal muscles contract, they not only expand the thorax but also extend the diaphragm muscle fibres to approach their optimal length and thus provide more muscle contraction force to resist the abdominal and pleural pressures during breathing. Therefore, representing this mechanism is important in order to simulate a mostly realistic mechanical environment of the chest during breathing.

The simulation results not only demonstrated the effectiveness of our approach to simulating the diaphragm motion but also illustrated the importance of simultaneous contraction of the diaphragm and inspiratory intercostal muscles when simulating human breathing.

### Validation of simulating the thorax deformation during normal quiet breathing by comparison with 4D-CT images

In normal quiet breathing, the diaphragm and intercostal muscles are the main inspiratory muscles, and the abdominal pressure is small compared with the diaphragmatic contracting force. Thus, we simulated the thorax deformation during normal quiet breathing by simultaneously activating the external intercostal muscles, parasternal intercostal muscles, and diaphragm. The obtained chest movement (front and lateral view in Fig. 8a) and diaphragm motion (oblique view in Fig. 8a) illustrate that the inspiratory chest movement was generated, and the dome of the diaphragm descended in the caudal-ventral direction. Even though the rib movement is small during normal quiet breathing, inspiratory rib motion can be observed in both the CT images and simulation results (Fig. 8c, d); the ribs slightly rotated cranially and ventrally as the circumference of the chest increased. This inspiratory rib motion also contained the bucket-handle and pump-handle motions as shown in Fig. 5.

Concerning the deformation of the diaphragm, we first compared the diaphragm shape after the inhaling state when the movements of the ribs and diaphragm reached their maximum during normal quiet breathing. The extracted CT images in Fig. 8c show that the diaphragm descended caudally and the shape remained unchanged, similar to the simulated diaphragm shape. The extracted CT images in Fig. 8d show that the posterior portion descended more caudally than the anterior portion. The same deformation pattern was also observed in the simulated diaphragm shape. The amount of movement on top of the diaphragmatic dome in the craniocaudal direction (MTDDCD) was approximately equal to 12 times that in the dorsal–ventral direction of the sternum in both the CT images and simulation results. The magnitude of the MTDDCD was 1.5 cm, which agrees with the measurements obtained by Wade during normal quiet breathing [30].

To confirm the diaphragm movement during the breathing process, we examined the time variation in the movement of the diaphragm by selecting three locations on plane Z of the diaphragm in 4D-CT (Fig. 8d). Similar time variations in the measured and simulated movements are given in Fig. 8e–g. The diaphragm movements extracted from the 4D-CT images were slower than the simulation results during both the inspiratory and expiratory procedures. This can be attributed to the viscosity of the airflow in the lungs during breathing because the redistribution of the airflow in the bronchi and alveoli throughout the lungs delays the response of the lung deformation to the inspiratory muscle contraction. Therefore, the deviation between the simulation and 4D-CT images indicates the importance of introducing a lung model and airway system which can represent the viscous effect in the chest during breathing.

By introducing the Hill-type transversely isotropic hyperelastic continuum skeletal muscle model and simultaneously activating the external intercostal muscles, parasternal intercostal muscles, and diaphragm, we were able to validate the effectiveness of the proposed approach for simulating the thorax deformation during normal quiet breathing by comparison with 4D-CT images.

## Conclusions

To simulate the thorax deformation driven by the diaphragm and intercostal muscles, we modelled the respiratory muscles according to continuum mechanics and by introducing muscle contractions. By introducing the Hill-type transversely isotropic hyperelastic continuum skeletal muscle model for the diaphragm and intercostal muscles, we were able to obtain the representative motions of the diaphragm and ribs as generated by muscle contractions.

For the ribs, inspiratory and expiratory rib movements including the representative bucket-handle and pump-handle motions were reproduced by activating the inspiratory and expiratory intercostal muscles individually. The rib rotation angles for the bucket-handle and pump-handle motions were validated by comparison to experimental measurements carried out by Troyer et al. [23]. For the diaphragm, we not only obtained the representative motion as observed in MRI scans but also demonstrated the interaction between the diaphragm and inspiratory intercostal muscles during breathing. The latter is important for simulating the mostly realistic mechanical environment of the chest during breathing. The effectiveness of the proposed approach at simulating the thorax deformation during normal quiet breathing was validated by comparison with 4D-CT images. A deviation between the simulation and clinical observation occurred in the rib and diaphragm motions; this was due to the lack of cervical accessory muscles in the model and the viscosity effect of the airflow in the lung.

This study was the first step towards establishing a computational mechanics model of the human respiratory system. Currently, we have established a chest wall FEM model which includes primary respiratory muscles but does not consider the influence of the abdominal wall and organs inside the chest. In future work, to establish an integrated computational biomechanics model of the human respiratory system, we will add auxiliary respiratory muscles and the organs in the abdominal wall and chest to the current model. We will also introduce an airway system and porous hyperelastic lung model to simulate the alveolar, pleural pressure, and lung volume change during human respiration. The final goal is to develop an effective tool for revealing the mechanisms of human respiration and ultimately for diagnosing and treating respiratory diseases.

## Abbreviations

RI: magnetic resonance imaging
4D-CT: four-dimensional computed tomography
CT: computed tomography
COPD: chronic obstructive pulmonary disease
MDCT: multi detector-row computed tomography
FEM: finite element method
PE: parallel element
SE: series element
CE: contractile element
MTDDCD: movement on top of the diaphragmatic dome in the craniocaudal direction

## References

[1] Matsuoka S, Kurihara Y, Yagihashi K, Hoshino M, Watanabe N, Nakajima Y (2008). Quantitative assessment of air trapping in chronic obstructive pulmonary disease using inspiratory and expiratory volumetric MDCT. Am J Roentgenol.

[2] Burrowes KS, Doel T, Brightling C (2014). Computational modeling of the obstructive lung diseases asthma and COPD. J Transl Med.

[3] Tian G, Hindle M, Lee S, Longest PW (2015). Validating CFD predictions of pharmaceutical aerosol deposition with in vivo data. Pharm Res.

[4] Manescu P, Ladjal H, Azencot J, Beuve M, Testa E, Shariat B (2014). Four-dimensional radiotherapeutic dose calculation using biomechanical respiratory motion description. Int J Comput Assist Radiol.

[5] Hall JE (2010). Guyton and Hall textbook of medical physiology.

[6] Loring SH (1992). Action of human respiratory muscles inferred from finite element analysis of rib cage. J Appl Physiol.

[7] Behr M, Pérès J, Llari M, Godio Y, Jammes Y, Brunet C (2010). A three-dimensional human trunk model for the analysis of respiratory mechanics. J Biomech Eng-T ASME.

[8] Ladjal H, Shariat B, Azencot J, Beuve M (2013). Appropriate biomechanics and kinematics modelling of the respiratory system: human diaphragm and thorax. IEEE Int Conf Intell Robet Syst.

[9] Ito H, Koshizuka S, Haga A, Nakagawa K (2011). Rib cage motion model construction based on patient-specific CT images between inhalation and exhalation. Med Imaging Technol.

[10] Ookura T, Ito H, Koshizuka S, Nomoto A, Haga A, Nakagawa K (2013). Diaphragm respiratory motion model with ribcage movement. Med Imaging Techol.

[11] Martins JAC, Pato MPM, Pires EB (2006). A finite element model of skeletal muscles. Virtual Phys Prototyp.

[12] Washio T, Okada J, Hisada T (2010). A parallel multilevel technique for solving the bidomain equation on a human heart with purkinje fibres and a torso model. SIAM Rev.

[13] Netter FH (2006). Atlas of human anatomy.

[14] De Troyer A, Boriek AM (2011). Mechanics of the respiratory muscles. Compr Physiol.

[15] Wriggers P (2006). Computational contact mechanics.

[16] Holzapfel DA (2000). Nonlinear solid mechanics.

[17] Farkas GA (1991). Mechanical properties of respiratory muscles in primates. Respir Physiol.

[18] Remmers JE (1970). Inhibition of inspiratory activity by intercostal muscle afferents. Respir Physiol.

[19] Martins PALS, Jorge RMN, Ferreira AJM (2006). A comparative study of several material models for prediction of hyperelastic properties: application to silicone-rubber and soft tissues. Strain.

[20] Pato MPM, Santos NJG, Areias P, Pires EB, De Carvalho M, Pinto S (2011). Finite element studies of the mechanical behaviour of the diaphragm in normal and pathological cases. Comput Methods Biomech Biomed Eng.

[21] De Troyer A, Legrand A (1995). Inhomogeneous activation of the parasternal intercostals during breathing. J Appl Physiol.

[22] Legrand A, De Troyer A (1999). Spatial distribution of external and internal intercostal activity in dogs. J Physiol.

[23] Wilson TA, Legrand A, Gevenois PA, De Troyer A (2001). Respiratory effects of the external and internal intercostal muscles in humans. J Physiol.

[24] Kolář P, Neuwirth J, Šanda J, Suchánek V, Svatá Z, Volejník J (2009). Analysis of diaphragm movement during tidal breathing and during its activation while breath holding using MRI synchronized with spirometry. Physiol Res.

[25] Cluzel P, Similowski T, Chartrand-Lefebvre C, Zelter M, Derenne JP, Grenier PA (2000). Diaphragm and chest wall: assessment of the inspiratory pump with MR imaging-preliminary observations. Radiology.

[26] Gauthier AP, Verbanck S, Estenne M, Segebarth C, Macklem PT, Paiva M (1994). Three-dimensional reconstruction of the in vivo human diaphragm shape at different lung volumes. J Appl Physiol.

[27] Cherniack NS, Haxhiu MA, Mitra J, Strohl K, Van Lunteren E (1984). Responses of upper airway, intercostal and diaphragm muscle activity to stimulation of oesophageal afferents in dogs. J Physiol.

[28] Dowling DJ, DiGiovanna EL, Schiowitz S, Dowling DJ (2005). Anatomic considerations of the thoracic cage. An osteopathic approach to diagnosis and treatment.

[29] DiMarco AF, Supinski GS, Budzinska K (1989). Inspiratory muscle interaction in the generation of changes in airway pressure. J Appl Physiol.

[30] Wade OL (1954). Movements of the thoracic cage and diaphragm in respiration. J Physiol.

